# Bringing Again Noble Metal Nanoparticles to the Forefront of Cancer Therapy

**DOI:** 10.3389/fbioe.2018.00143

**Published:** 2018-10-08

**Authors:** Ylea Vlamidis, Valerio Voliani

**Affiliations:** Center for Nanotechnology Innovation, Istituto Italiano di Tecnologia, Pisa, Italy

**Keywords:** persistence, oncology, nanoparticles, theranostic agent, clinical translation, nanomedicine, nanobiotechnology

## Abstract

Nanomaterials have attracted increasing interest for their potentiality to revolutionize the diagnosis and treatment of many diseases, especially neoplasms. Interestingly, there is a huge imbalance between the number of proposed nanoplatforms and the few ones approved for clinical applications. This disequilibrium affects in particular noble metal nanoparticles (NPs), that present no-approved platform and very few candidates in clinical trials because of the issue of persistence. In this perspective, we discuss if nanomedicine is generally keeping its promises with a focus on the approach that could fill the gap between NPs and oncology in the next future: the ultrasmall-in-nano.

## The lack of translation of noble metal nanoparticles

Nanoplatforms, due to their unique size/shape-influenced chemical, physical, and physiological features, are widespread studied for applications in many fields of academic research. In particular, they should have pushed forward the paradigms in key-sectors such as energy harvesting, environmental remediation, and medicine. About the latter, researchers have profused most of their efforts toward the development of novel or enhanced diagnosis and treatments of neoplasms (Lee et al., 2013; Yamada et al., 2015; Yang et al., 2015; Abadeer and Murphy, 2016). After more than 20 years from the approbation of the first nanomedicine, Doxil—doxorubicin-loaded PEGylated nano-liposomes (Barenholz, 2012)—, the question is: has nanomedicine kept its promises in oncology?

At a glance, the answer is “in part.” Indeed, after Doxil, regulatory agencies such as FDA and EMA have approved other 50 “nanodrugs,” about other 30 products are undergoing clinical trials and some of them could be on the market in the next future (Anselmo and Mitragotri, 2016; Bobo et al., 2016; Caster et al., 2017). Approved nanoplatforms are mainly composed by lipids (such as Myocet, which is commercially available to treat metastatic breast cancer) or proteins (e.g., Abraxane, which is employed to treat different types of cancer), for a US billions market (Ragelle et al., 2017). In particular, it is expected that the global market of nanopharmaceuticals will reach $412 billions in 2019 (Ragelle et al., 2017); a market in which cancer therapeutic products such as Abraxane and Doxil play a pivotal role. The enhanced action is commonly related to the size and, in some cases, to the ability to make commonly employed drugs more available to the action site(s) (Ragelle et al., 2017). This means that the full potentiality of nanomaterials to revolutionize the clinical practice in oncology is yet not reached (Chen et al., 2017). Remarkably, despite a number of appealing features to design efficient and less invasive cancer treatments (Dreaden et al., 2012; Sun et al., 2014; Yang et al., 2015), noble metal nanoparticles (NPs) are still on the bench-side. Such NPs based on gold, silver, or platinum are widespread investigated in medical applications due to their size and shape dependent optoelectronic properties. In particular, Au NPs have been commonly proposed for cancer therapy and diagnostics, Ag NPs displays interesting antibacterial activity, whereas Pt NPs can be employed as reactive oxygen species scavengers, due to their ability to catalyze biological reactions. The most common potential applications of noble metal NPs in medicine are summarized in Table 1.

**Table 1:** Main medical applications of noble metal NPs in academic studies.

**Type of metal NPs**	**Biomedical applications**	**References**
Au NPs	Cancer treatment	Dreaden et al., 2012
	Diagnostic/imaging	Abadeer and Murphy, 2016
	Osteoinductive agent during dental implant therapy	Jadhav et al., 2018
Pt NPs	Cancer treatment	Porcel et al., 2010
	Diagnostic/imaging	Liu et al., 2011
	Scavengers of reactive oxygen species	Moglianetti et al., 2016
	Improve dentin bond strength	Hoshika et al., 2010
Ag NPs	Cancer treatment	Ge et al., 2014
	Antibacterial activity	Li et al., 2010
	Antifungal activity	Gopinath and Velusamy, 2013
	Antiviral activity	Xiang et al., 2011
	Dental resin filler composite	Abbasi et al., 2014

It is worth to notice that the lack of translation of noble metal NPs to clinics is mainly related to the issue of persistence (Etheridge et al., 2013; Ehlerding et al., 2015; Bobo et al., 2016).

The persistence of NPs in organisms leads to possible interference with common medical diagnoses and can induce severe damages and gene expression alterations (Khlebtsov and Dykman, 2011; Cassano et al., 2018). Regulatory agencies require a complete clearance from the body of all the pharmaceutical and their components in an acceptable timeframe (Zamboni et al., 2012; Eaton et al., 2015; Sainz et al., 2015). Therefore, NPs have to be designed with proper size, shape, charge, and surface chemistry, in order to ensure the optimal conjugation between action and clearance. The excretion is an essential biological process to eliminate materials from organisms, and can be accomplished by renal or hepatic pathways (Liu et al., 2013). The renal pathway relies on glomerular filtration in kidneys, which size threshold is typically <8 nm, denoting that only materials with ultrasmall hydrodynamic diameters (HD) can be efficiently eliminated through the urinary system (Du et al., 2017; Wang and Liu, 2018). On the other hand, materials with HD >8 nm (and generally metal NPs with intriguing behaviors for theranostic applications show HD >8 nm) are mainly captured by liver and spleen (Tsoi et al., 2016). If captured materials are biodegradable, the building blocks are then excreted by bile and feces (Sun et al., 2014; Tsoi et al., 2016). Metal NPs are not easily (bio)degradable from organisms, resulting in their accumulation in the reticuloendothelial system (RES), especially in liver and spleen, for long time after the theranostic action (Khlebtsov and Dykman, 2011; Ali et al., 2017). In general, NPs are internalized by cells through endocytic mechanisms and may be exposed to enzymes comprised in lysosomes. Such agents are able to mediate the degradation/corrosion of metal NPs. In particular, the acidic environment of the vesicles can lead to etching of the NPs, causing the release of relatively toxic free metal ions, as for example Ag^+^ and Au^+^/^3+^. Such ions can potentially affect cell homeostasis and functions, causing cytotoxicity (Deng and Gao, 2016). Obviously, the long-term etching leads to a decrease of the NPs diameter, allowing their long-term gradual elimination from the body (Sabella et al., 2014). Overall, kidney passive filtration represents the most reliable excretion route for metal NPs in order to minimize potential side effects resulting from long-term metal persistence (Zarschler et al., 2016; Cassano et al., 2018).

As a first attempt to overcome the issue of persistence, many efforts have been done on the reduction of the size of NPs to ultrasmall range (<8 nm, ultrasmall nanoparticles, USNPs) in order to enhance their renal clearance efficiency (Zarschler et al., 2016). Due to this smart approach an interesting level of metal excretion was reached (Zarschler et al., 2016; Schmid et al., 2017), but commonly losing some key-features of NPs, among which physical and physiological behaviors (e.g., localized surface plasmon resonances and passive accumulation in targets; Cassano et al., 2018). It is worth to remember here that USNPs can exhibit cytotoxic profile when the stabilizing ligands allow for direct access to the metal surface either for the direct interaction with biomolecules or for catalytic activity of the unshielded metal surface; this topic is carefully discussed elsewhere (Schmid et al., 2017).

A groundbreaking advance to bring again NPs to the forefront of cancer theranostics relies on the ultrasmall-in-nano approach for the design of nanoplatforms able to jointly combine the unique behaviors of plasmonic NPs with the body excretion by the renal pathway (Cassano et al., 2018).

Ultrasmall-in-nano platforms are structures usually composed by aggregates of plasmonic USNPs comprised in (bio)degradable nanocapsules. A significant example in this direction are the nature-inspired passion fruit-like nano-architectures (NAs) (Cassano et al., 2015, 2017). NAs are produced by an extreme versatile and robust synthetic protocol (Cassano et al., 2017; Pocovi-Martinez et al., 2018). NAs biodegrade in <48 h to polymers, endogenous-glutathione (GSH) coated plasmonic gold USNPs and silicic acid (Cassano et al., 2016; Avigo et al., 2017). The remarkable biocompatibility of NAs and of the relative building blocks was assessed in cultured cells and Zebrafish models (Cassano et al., 2016; d'Amora et al., 2018). Furthermore, some of their potential applications as drug delivery vehicles and imaging contrast agents were already demonstrated, pointing out that all the components of NAs are pivotal (Cassano et al., 2016; Avigo et al., 2017; Armanetti et al., 2018; Mapanao et al., 2018).

Due to the peculiar structure of ultrasmall-in-nano platforms, toxicity issues related to long-term accumulation in organs are overcome, unlocking the true potential of noble metal nanotheranostics for cancer treatment and beyond.

Nevertheless, it is interesting to notice that despite the issue of persistence is well-known, there are still an increasing number of publications, also in top tiers Journals, about the design of non-excretable metal nanoplatforms with intriguing features and increasingly complex structure—meaning a number of, for example, imaging and therapeutic applications on the same platform—, but without any real potentiality of translation. At this point the question is: if this kind of NPs cannot be translated to clinics, why is there this trend? Indeed, the main interesting behaviors of metal NPs are now well described together with their potential applications as innovative or synergistic nanoplatforms for theranostics. Moreover, more complex is a nanoplatform and higher will be its intrinsic cost (i.e., market potentiality is strongly reduced; Malinoski, 2014).

The context described together with the lack of an “industrial culture” of many researchers help to explain the huge imbalance between the metal NPs proposed on peer-reviewed journals and the very few candidates in clinical trials (listed in Table 2). Besides basic research, which has to be always encouraged, an ideal approach to hold the promises of nanomedicine in the very next future should be to: (i) develop the most promising leading candidates that overcome significant key-issues (such as toxicity and persistence in organism after the medical action), and (ii) design novel NPs with the aim to overcome a specific issue, in order to focus on translational research.

**Table 2:** List of the principal nanomedicines based on metal NPs in clinical trials (source: ClinicalTrials.gov).

**Name**	**Nanomedicine**	**NPs diameter (nm)**	**Indication**	**Status**
AuroLase	PEG-coated silica-gold nanoshells	1-3	Primary and/ or metastatic lung tumors	Phase I
AGulX	Gadolinium-based NPs	3	Brain metastases	Phase I
Magnablate	Magnetic iron NPs	Not available	Prostate cancer	Phase I
Clariscan	Iron Oxide NPs	5–7	Enhanced MRI Contrast	Phase III
NBTXR3	Hafnium oxide NPs	50	Locally advanced squamous cell carcinoma	Phase I/II
–	Silver NPs	12–15	Inflammatory diseases	Phase I

## General remarks

As pointed out in the previous section, the market of nanoparticles for clinical applications should increase from 15% of the total pharmaceutical market in 2014 to 22% in 2019 mainly thanks to the release of nanoplatforms that have increased the efficiency of well-known drugs (Ragelle et al., 2017). On the other hand, the next revolution in oncology has still to come, and it will probably relate to the release of ultrasmall-in-nano noble metal nanoparticles, which will be able to jointly combine innovative, synergistic and non-invasive treatments for a number of neoplasms avoiding the issue of metal persistence in the organism.

Nevertheless, some other topics have to be considered while designing a nanoplatform. For example, it is pivotal to have a deep understanding of the physicochemical properties of the materials, and to develop reasonable, safe, reproducible and scalable synthesis procedures for the production of sterilized products. These subjects are comprehensively discussed elsewhere (Zamboni et al., 2012; Sainz et al., 2015). In order to accelerate the transition of basic nanoscale particles and devices into clinical applications, some organizations have recently emerged, such as the Nanotechnology Characterization Laboratory (NCL) in US and the Nanomedicine Characterization Laboratory (EUNCL) in Europe. These organizations provide guidelines for the development and characterizations of nanotheranostics together with a support to researchers by a free preclinical characterization, pharmacokinetic, efficacy, and toxicity testing of promising nanotech cancer therapeutics and diagnostics by employing a standardized “Assay Cascade.” A peculiar assay cascade for nanomaterials is obviously desirable, as the structural complexity of NPs makes the regulation guidelines for small molecules somehow inadequate and insufficient. For example, the features of NPs can be easily altered not only by slight changes in raw materials, but also by small modifications in manufacturing processes, that can result in limited alterations of the structure but in significantly altered biological and biodistribution behaviors. Furthermore, it would also be desirable the development of peculiar diagnostic methods/probes able to follow nanotheranostics effects without interacting nor interfering with the nanoplatforms. Indeed, therapeutic response monitoring should provide feedback that can guide follow-up regimens. Another aspect, which has yet to be fully understood, is related to the responsivity of neoplasms, which can also significantly change between patients. Considering this aspect, a complete preclinical evaluation of the behaviors of nanoplatforms (safety toxicology and absorption/distribution/metabolism/excretion—ADME package) should be obtained in a couple of *in vivo* models.

A quite underestimated topic related to the employment of metal NPs in clinical practice is the environmental safety assessment. Since 2011, the European Commission is focusing on the risks related to the fate of nanomaterials in the air, water, waste, industrial emissions, to provide more insight for further legislative guidance and risk assessment. Indeed, there is still a considerable lack of data on both the exposure to nanomaterials via the environment and to the exposure of environment to human-made nanomaterials. Consequently, no specific provisions for nanomaterials employment have yet been established by EU legislation (Second Regulatory Review on Nanomaterials COM/2012/0572). The EU Commission is currently working on detection and monitoring methods for nanomaterials and their validation to ensure the proper implementation of the norms.

## Conclusion

Overall, in order to promote the translation of the new findings to patients, extended collaborations involving scientists from the Health Agencies as well as academic, clinicians, and industry scientists would be fundamental. Besides an extended collaboration, it would be worth to establish a transnational coordination and funding in a flagship framework fashion. Last but not least, researchers should: (i) employ a part of their time to disseminate science and their results to the community and to students in order to increase the science awareness in the society, and (ii) avoid to overemphasize their findings in peer-reviewed manuscripts, as such practice may discourage public confidence in nanomedicine.

## Author contributions

YV and VV have written the manuscript and are familiar with its content.

### Conflict of interest statement

The authors declare that the research was conducted in the absence of any commercial or financial relationships that could be construed as a potential conflict of interest.
